# Supplementary material for: Radioembolization-Induced Changes in Hepatic [18F]FDG Metabolism in Non-Tumorous Liver Parenchyma
Source: Diagnostics (Basel). 2022 Oct 17;12(10):2518. doi: 10.3390/diagnostics12102518 (PMC9600277; doi:10.3390/diagnostics12102518)
Supplement: Supplementary file 1 [file diagnostics-12-02518-s001.zip › diagnostics-1961023-supplementary.pdf]

Supplementary Table S1. PERCIST criteria.

	PERCIST
**Main criteria**	
Patient preparations	4-6 hours fasting
Serum glucose level	< 200 mg/dl (<11,1 mmol/l)
Injected activity	Center dependent, but within 20% difference between scans
Measurement unit	1. SUV_{LBM} = SUL<br>2. SUL_{peak} = 1 cm^3 VOI
Glucose correction	No
Timing restrictions	PET at least 10 days after last chemotherapy<br>8-12 weeks after external beam radiotherapy
Scan restrictions	>50 minutes p.i.
	< 15 min difference in p.i. times between studies. *(but always > 50 min p.i.)*
	**Normal liver SUL must be within 20% (and < 0,3 SUL units) between baseline and follow-up scans**
	Always same scanner, same model, same reconstructions
Partial volume correction	No
Correlation with other tissues *	3 cm diameter VOI in right liver lobe, expressed in SUL_{mean} + SD.<br>If liver is affected or VOI cannot be placed: 1 cm diameter axial (ROI) with 2 cm diameter in z-axis (= ellipse VOI)
**Measurements**	
Tumor	SUL_{peak} VOI centered around hottest point in tumor foci (around maximum SUL pixel; which also should be noted)
	Most intense hypermetabolic tumors
Number of lesions	Up to 5 lesions (up to 2 per organ)
	2 cm in diameter (though smaller lesions with sufficient uptake might be included).
	Baseline tumor uptake: > 1,5 x $SUL_{mean,liver}$ + 2 SDs $SUL_{mean,liver}$<br>*Or* > 2 x $SUL_{mean,bloodpool}$ + 2 SDs $SUL_{mean,mediastinum}$
Follow up measurements	Most intense lesions, not necessarily the same lesions
Additional parameters	

Definition response:

Calculation	Sum of PERCIST lesions
Complete metabolic response	Visual disappearance of all metabolic active tumors
Partial metabolic response	>30% reduction and at least 0,8 unit decline in SUL_{peak}
Stable disease	Between partial response and progressive disease
Progressive metabolic disease	>30% increase and at least 0,8 unit increase in SUL_{peak} *or* new metabolic active tumors *or* >75% increase in TLG

Legend: SUV = standardized uptake value, LBM = lean body mass, SUL = SUV corrected for lean body mass, p.i. = post-injection, SD = Standard deviation, TLG = total lesion glycolysis

** modified PERCIST 1.0: threshold for minimal metabolically measurable tumor activity is 1.5 x mean liver SUL instead of 1.5 x mean liver SUL + 2 SDs (as in the conventional PERCIST)*
